# Clinicopathological characteristics and survival outcomes of male breast cancer according to race: A SEER population-based study

**DOI:** 10.18632/oncotarget.18265

**Published:** 2017-05-26

**Authors:** He-Fen Sun, Yang Zhao, Shui-Ping Gao, Liang-Dong Li, Wen-Yan Fu, Hong-lin Jiang, Meng-Ting Chen, Li-Peng Yang, Wei Jin

**Affiliations:** ^1^ Department of Breast Surgery, Key Laboratory of Breast Cancer in Shanghai, Collaborative Innovation Center of Cancer Medicine, Fudan University Shanghai Cancer Center, Shanghai, 200030, China; ^2^ Department of Oncology, Shanghai Medical College, Fudan University, Shanghai, 200030, China; ^3^ Division of Molecular Medicine and Genetic, Department of Internal Medicine and Life Sciences Insititute, University of Michigan, Ann Abor, Michgan 48109, USA; ^4^ Department of pathology, School of Basic Medical Sciences, Fudan University, Shanghai, 200030, China

**Keywords:** male breast cancer, race, overall survival, SEER

## Abstract

To investigate the clinicopathological characteristics and survival outcomes of breast cancer in the male population, 8,607 cases of patients were identified in the Surveillance, Epidemiology, and End Results (SEER) database, including white males (*n* = 7122), black males (*n* = 1111), and other males (American Indian/AK Native, Asian/Pacific Islander) (*n* = 374). Black male breast cancer patients were more likely to be in stages II–IV and have more advanced tumors. The rate of lymph node (LN) involvement at diagnosis was higher in black men than in whites and others. The ER- and PR-positive rates were lower in black men than in whites and others. The distant metastasis rate was higher in blacks than in whites and others. Furthermore, the overall survival (OR) rates and breast cancer-specific survival rates were significantly poorer in blacks than in whites and others (χ^2^ = 29.974, *P* < 0.001; χ^2^ = 7.285, *P* = 0.026, respectively). In a multivariate analysis, the results showed that race could also be a prognostic indicator (*P* < 0.001). Moreover, significant differences were also observed in OS among 1:1:1 matched white, black, and other groups (*P* < 0.001). Differences in outcomes may be partially explained by differences in tumor grades, LN status, and ER and PR status between the 3 groups. This study might provide insights into a better understanding of male breast cancer.

## INTRODUCTION

Male breast carcinoma (MBC) is a rare disease with steady incidence rates; it comprises about 1% of all cancers in men in Western countries [1–2]. Although rare, breast cancer (BC) affects men’s health and quality of life. The present study showed that the mean age of MBC patients was about 60–65 years old. However, this disease may develop in a wide range of ages. For example, the youngest MBC patient was 9 years old and the oldest was above 90 years [3].

As a result of the absence of screening programs in men, MBCs are usually diagnosed at a more advanced age. In the SEER data, the median ages at diagnosis of breast cancer were 67 and 62 years in males and females, respectively [4]. MBC patients are also diagnosed with a more severe clinical manifestation with relatively larger tumor sizes and more frequent lymph node involvement than female breast cancer (FBC) patients [5]. Moreover, male breast cancer also develops with a much higher proportion of positive tumor hormone receptors, a significantly prolonged treatment delay, and a more advanced tumor, node, and metastasis (TNM) stage of the disease at the time of diagnosis than FBC [6]. There are some differences in clinical and biological characteristics between FBC and MBC. However, the treatment of MBC is currently based on FBC due to the inadequate characterization [7–8].

Although the incidence of MBC is lower than that of FBC, a substantial variable may exist between different countries. The incidence of MBC in Thailand (0.14 per 100 000 man-years) was significantly lower than that in Israel (1.08 per 100 000 man-years). The variability in rates may be due to population-specific factors [9].

In specific population groups, cancer disparities exist in the incidence, prevalence, mortality, and burden of cancer and related adverse health conditions [10]. Of all the disparities, the differences in cancer related to race and ethnicity have been well described and are major public health concerns. For example, black men have higher incidence and death rates than white men when considering all cancer sites combined; black women also have higher death rates than white women [11–13]. These disparities apply to much of the United States, where whites and blacks are the predominant racial groups. However, a majority of studies ignore other races, including American Indians, AK Natives, Asians, and Pacific Islanders. Therefore, we wanted to know whether there was also some variability in male breast cancer in different races. Therefore, the aim of this study is to report clinicopathological characteristics and outcomes of a series of MBCs in different races.

## RESULTS

### Clinical characteristics of the study population

Overall, 8,607 patients with male breast cancer were enrolled, including 7,122 white patients, 1,111 black patients, and 374 patients of other races (including American Indians/AK Natives and Asian/Pacific Islanders). Their characteristics were analyzed and the results are summarized in Table 1. There were significant differences in clinical characteristics, including the year of diagnosis, age, tumor size, LN status, AJCC stage, ER status, PR status, and HER2 status. Among the 3 populations, white patients presented with an older age (50–85 years: 91.2% vs. 85.0% and 86.6%, respectively; *P* < 0.001). Furthermore, black MBC patients were more likely to be stages II-IV (9.7% vs. 8.3% and 6.7% in stage II, 4.7% vs. 3.3% and 3.2% in stage III, 3.4% vs. 1.5% and 1.9% in stage IV, respectively; *P* < 0.001) and to have more advanced tumors (2 cm < tumor size ≤ 5 cm: 23.0% vs. 20.8% and 19.5%, tumor size > 5 cm: 11.2% vs. 6.2% and 7.2%, respectively; *P* < 0.001). In addition, the rate of LN involvement at diagnosis was higher in blacks than in whites and others (29.6% vs. 22.9% and 23.3%, respectively; *P* < 0.001). An ER-positive rate was detected in 66.6% of the whites, 65.7% of the blacks, and 70.3% of the others (*P* < 0.001). Similarly, PR was expressed as 58.9%, 54.4%, and 66% of the whites, blacks, and others, respectively (*P* < 0.001). HER2 positivity was higher in the blacks than in the others and the whites (3.4% vs 2.1% and 2.1%, respectively; *P* = 0.002). The incidence of distant metastasis was higher in blacks than in the whites and others (bone metastasis: 2.3% vs 1.0 vs 1.1, *P* < 0.001; brain metastasis: 0.2 vs 0.1 vs 0, *P* = 0.010; liver metastasis: 0.5 vs 0.2 vs 0.3, *P* = 0.002; lung metastasis; 1.5 vs 0.6 vs 1.1, *P* < 0.001).

**Table 1:** Patient characteristics in white patients compared to blacks and others

Variables	White		Black		other^a^		Total		
	*n* = 7122		*n* = 1111		*n* = 374		*n* = 8607		
	No.	%	No.	%	No.	%	No.	%	*p*
Median follow-up (months) (IQR)	115 (111–119)		105 (93–116)		91 (78–103)				
Year of diagnosis									
1973–1993	1553	21.8	189	17.0	65	17.4	1807	21.0	**< 0.001**
1994–2013	5569	78.2	922	83.0	309	82.6	6800	79.0	
age (years)									
10–49	630	8.8	167	15.0	50	13.4	847	9.8	**< 0.001**
50–85	6492	91.2	944	85.0	324	86.6	7760	90.2	
Laterality									
right	3375	47.4	540	48.6	178	47.6	4093	47.6	0.346
left	3640	51.1	560	50.4	187	50.0	4387	51.0	
bilateral	107	1.5	11	1.0	9	2.4	127	1.5	
Grade									
I	703	9.9	106	9.5	34	9.1	843	9.8	0.385
II	2795	39.2	414	37.3	148	39.6	3357	39.0	
III	1966	27.6	337	30.3	107	28.6	2410	28.0	
IV	109	1.5	13	1.2	10	2.7	132	1.5	
unknown	1549	21.7	241	21.7	75	20.1	1865	21.7	
AJCC stage									
I	532	7.5	85	7.7	29	7.8	646	7.5	**< 0.001**
II	592	8.3	108	9.7	25	6.7	725	8.4	
III	238	3.3	52	4.7	12	3.2	302	3.5	
IV	108	1.5	38	3.4	7	1.9	153	1.8	
unknown	5652	79.4	828	74.5	301	80.5	6781	78.8	
Tumor size (cm)									
≤ 2	1656	23.3	224	20.2	105	28.1	1985	23.1	**< 0.001**
> 2 and ≤ 5	1484	20.8	255	23.0	73	19.5	1812	21.1	
> 5	441	6.2	124	11.2	27	7.2	592	6.9	
unknown	3541	49.7	508	45.7	169	45.2	4218	49	
LN status									
Negative	1952	27.4	274	24.7	118	31.6	2344	27.2	**< 0.001**
Positive	1629	22.9	329	29.6	87	23.3	2045	23.8	
To be continued									
unknown	3541	49.7	508	45.7	169	45.2	4218	49.0	
ER									
Negative	233	3.3	62	5.6	21	5.6	316	3.7	**< 0.001**
Positive	4745	66.6	730	65.7	263	70.3	5738	66.7	
unknown	2144	30.1	319	28.7	90	24.1	2553	29.7	
PR									
Negative	678	9.5	172	15.5	32	8.6	882	10.2	**< 0.001**
Positive	4192	58.9	604	54.4	247	66	5043	58.6	
unknown	2252	31.6	335	30.2	95	25.4	2682	31.2	
HER2									
Negative	1219	17.1	227	20.4	61	16.3	1507	17.5	**0.002**
Positive	147	2.1	38	3.4	8	2.1	193	2.2	
unknown	5756	80.8	846	76.1	305	81.6	6907	80.2	
Radiation									
Yes	1612	22.6	268	24.1	75	20.1	1955	22.7	0.195
No	5424	76.2	835	75.2	292	78.1	6551	76.1	
unknown	86	1.2	8	0.7	7	0.1	101	1.2	
Bone metastasis									
No	1434	20.1	264	23.8	72	19.3	1770	20.6	**< 0.001**
yes	74	1.0	25	2.3	4	1.1	103	1.2	
unknown	5614	78.8	822	74.0	298	79.7	6734	78.2	
Brain metastasis									
No	1497	21.0	285	25.7	76	20.3	1858	21.6	0.010
Yes	10	0.1	2	0.2	0	0	12	0.1	
unknown	5615	78.8	824	74.2	298	79.7	6737	78.3	
Liver metastasis									
No	1493	21.0	283	25.5	75	20.1	1851	21.5	0.002
Yes	14	0.2	6	0.5	1	0.3	21	0.2	
unknown	5615	78.8	822	74.0	298	79.7	6735	78.3	
Lung metastasis									
No	1462	20.5	271	24.4	72	19.3	1805	21.0	**< 0.001**
Yes	42	0.6	17	1.5	4	1.1	63	0.7	
unknown	5618	78.9	823	74.1	298	79.7	6739	78.3	

*P*-value was calculated among all groups by the Chi-square test, and a bold type indicates significance. a: Including American Indian/Alaskan native, Asian/Pacific Islander and others-unspecified.

### Comparison of MBC survival among whites, blacks, and other races

**Figure 1:**
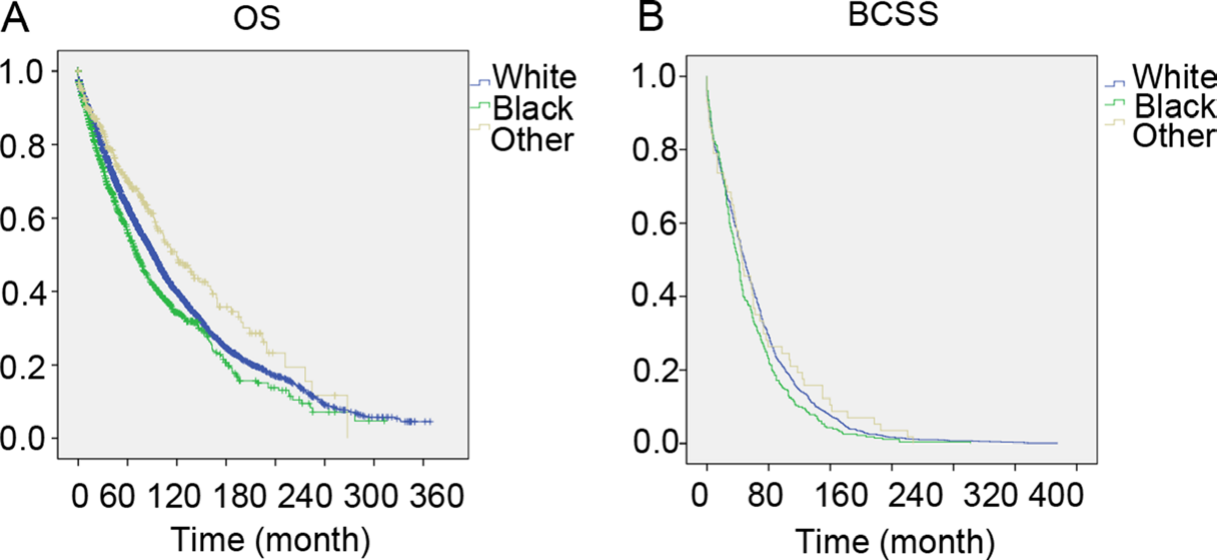
The overall survival and breast cancer specific survival of White, Black and other patients Kaplan meier test for overall survival (χ^2^ = 29.974, *P* < 0.001) (Figure 1A) and breast cancer specific survival (χ^2^ = 7.285, *P* = 0.026, Figure 1B) to compare White patients to Blacks and others.

As shown in Kaplan-Meier plots, 30-year overall survival (OS) was better in other patients than in the white and black populations (χ ^2^ = 29.974, *P* < 0.001, Figure 1A). We also analyzed the breast cancer-specific survival (BCSS) and slightly significant differences were observed (χ ^2^ = 7.285, *P* = 0.026, Figure 1B). The median survival time was 102 months (95% CI: 98–1105), 80 months (95% CI: 72–88), and 133 months (95% CI: 108–157) in the white, black, and other patients, respectively. The 30-year OS likely represents mortality from other causes; we capped it at 15 years, and it showed similar results in the Supplementary Figure 1. Furthermore, we used the Cox proportional hazards model to investigate the effects of the clinical characteristics on OS (Table 2). Many prognostic indicators were also found to be significantly associated with OS in the univariate analysis, including the year of diagnosis, laterality, tumor grade, tumor size, age, LN status, ER status, PR status, HER2 status, and radiation (Table 2). The results showed that race could also be a prognostic indicator. Taking the white race as the reference, we found that the white race could be a protective factor when compared to the black race (HR = 1.208, 95% CI: 1.107–1.319, *P* < 0.001), but could also be a risk factor when compared to the others (HR = 0.775, 95% CI: 0.659–0.911, *P* = 0.002). All the variables were included in the multivariate analysis to estimate the prognostic factors that were identified in the univariate analysis (Table 2). Race was also an independent prognostic factor in the multivariate analysis after adding the other prognostic factors. When we adjusted for white patients as a control group, the white race could also be an independent protective factor when compared to the black race (HR = 1.208, 95% CI: 1.106–1.320, *P* < 0.001), while it was a risk factor when compared with the other races (HR = 0.801, 95% CI: 0.681–0.942, *P* = 0.007).

**Table 2:** Univariate and multivariate analysis of overall survival (OS)

Variables	Univariate analysis		Multivariate analysis	
	**HR (95% CI)**	***P*–Value**	**HR (95% CI)**	***P*–Value**
Year of diagnosis				
1973–1993	reference		reference	
1994–2013	0.861 (0.087–0.920)	**< 0.001**	1.002 (0.917–1.095)	0.963
age (years)				
10–49	reference		reference	
50–85	2.373 (2.105–2.676)	**< 0.001**	2.494 (2.211–2.814)	**< 0.001**
Laterality				
right	reference		reference	
left	1.004 (0.946–1.066)	0.885	1.000 (0.942–1.061)	1.000
bilateral	0.448 (0.353–0.568)	**< 0.001**	1.620 (1.262–2.079)	**0.001**
Grade				
I	reference		reference	
II	1.313 (1.159–1.487)	**< 0.001**	1.267 (1.118–1.436)	**< 0.001**
III	1.712 (1.508–1.943)	**< 0.001**	1.559 (1.372–1.772)	**< 0.001**
IV	1.988 (1.585–2.494))	**< 0.001**	1.759 (1.399–2.211)	**< 0.001**
unknown	1.736 (1.555–1.999)	**< 0.001**	1.478 (1.293–1.690)	**< 0.001**
AJCC stage				
I	reference		reference	
II	1.735 (1.195–2.520)	**0.004**	0.940 (0.636–1.389)	0.755
III	2.361 (1.561–3.570)	**< 0.001**	1.070 (0.694–1.650)	0.758
IV	8.156 (5.489–12.119)	**< 0.001**	2.840 (1.863–4.329)	**< 0.001**
unknown	2.562 (1.892–3.469)	**< 0.001**	0.791 (0.524–1.192)	0.262
Tumor size (cm)				
≤ 2	reference		reference	
> 2 and ≤ 5	1.995 (1.756–2.266)	**< 0.001**	1.802 (1.574–2.063)	**< 0.001**
> 5	3.334 (2.850–3.899)	**< 0.001**	2.519 (2.132–2.977)	**< 0.001**
unknown	1.779 (1.595–1.984)	**< 0.001**	1.630 (1.432–1.856)	**< 0.001**
LN status				
Negative	reference		reference	
Positive	1.584 (1.417–1.770)	**< 0.001**	1.261 (1.122–1.418)	**< 0.001**
unknown	1.371 (1.249–1.506)	**< 0.001**	–	–
ER				
Negative	reference		reference	
Positive	0.684 (0.587–0.796)	**< 0.001**	0.800 (0.675–0.964)	**0.010**
unknown	0.892 (0.765–1.040)	0.144	1.211 (0.912–1.608)	0.186
Continued				
PR				
Negative	reference		reference	
To be continued				
Positive	0.763 (0.689–0.845)	**< 0.001**	0.861 (0.768–0.964)	**0.010**
unknown	0.988 (0.891–1.095)	0.813	0.675 (0.524–0.870)	**0.002**
Her2				
Negative	reference		reference	
Positive	1.698 (1.166–2.473)	**0.006**	1.305 (0.894–1.905)	0.168
unknown	1.606 (1.361–1.894)	**< 0.001**	2.059 (1.484–2.855)	**< 0.001**
Radiation				
Yes	reference		–	–
No	0.950 (0.884–1.019)	0.154	–	–
unknown	1.397 (0.977–1.998)	0.067	–	–
Race				
White	reference		reference	
Black	1.208 (1.107–1.319)	**< 0.001**	1.208 (1.106–1.320)	**< 0.001**
Other^a^	0.775 (0.659–0.911)	**0.002**	0.801 (0.681–0.942)	**0.007**

The CI and *P*-value was calculated by Cox proportional hazards model and the bold type indicates significance. a:Including American Indian/Alaskan native, Asian/Pacific Islander and others-unspecified.

### Survival analysis in matched groups

**Figure 2:**
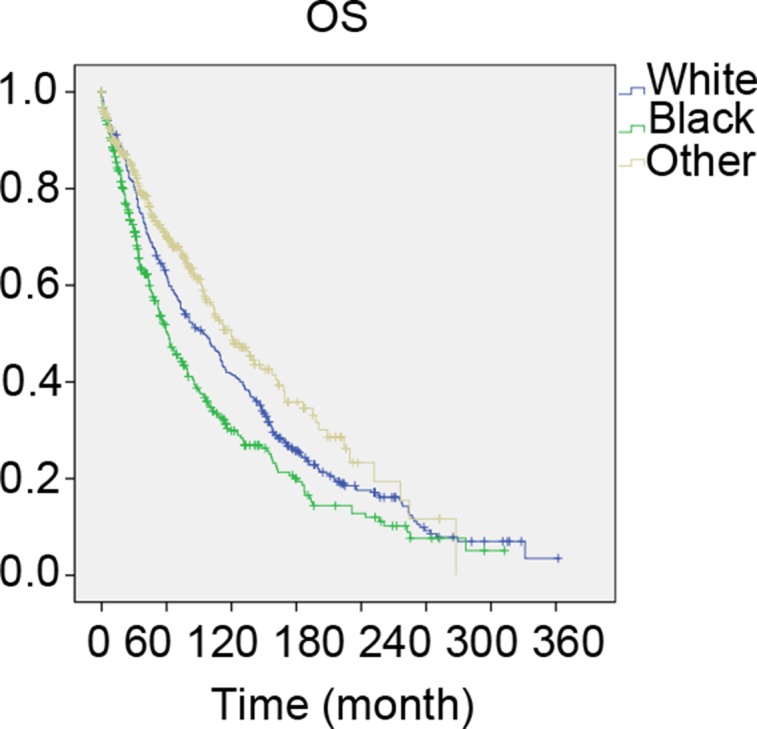
The overall survival of 1:1:1 matched groups of White, Black and other patients Kaplan meier test for overall survival of 1:1:1 matched groups to compare to compare white patients to Blacks and others (χ^2^ = 26.811, *P* < 0.001).

There was a large difference among the 3 races. To ensure that the outcomes were not based on the differences of patient quantity of the groups, we performed a 1:1:1 (white: black: other) matched case control analysis using the propensity score matching method. We finally focused on a group of 1122 patients, including 374 patients in each racial type (Table 3). Compared to the results in Table 1, similar results are shown in Table 3. There were also significant differences in the clinical characteristics, including tumor size, LN status, AJCC stage, ER status, and PR status, except in age and laterality. Furthermore, we also found that the black race was associated with a poorer prognosis in OS, similar to the total group (χ^2^ = 26.811, *P* < 0.001, Figure 2).

**Table 3:** Patient Characteristics in the 1:1 matched groups

Variables		white		black		**other**^a^		total		
	*n* = 374		*n* = 374		*n* = 374		*n* = 1122			
	No.	%	No.	%	No.	%	No.	%	*p*	
Median follow-up (months) (IQR)		290(255–324)		148(128–167)		91(78–103)				
Year of diagnosis										
1973-1993		263	70.3	141	37.7	65	17.4	469	41.8	**< 0.001**
1994-2013		111	29.7	233	62.3	309	82.6	653	58.2	
age (years)										
10-49		37	9.9	51	13.6	50	13.4	138	12.3	0.22
50-85		337	90.1	323	86.4	324	86.6	984	87.7	
Laterality										
right		189	50.5	182	48.7	178	47.6	549	48.9	0.373
left		174	46.5	188	50.3	187	50.0	549	48.9	
bilateral		11	1	4	0.4	9	0.8	24	2.1	
Grade										
I		36	9.6	19	5.1	34	9.1	89	7.9	**< 0.001**
II		95	25.4	101	27.0	148	39.6	344	30.7	
III		70	18.7	120	32.1	107	28.6	297	26.5	
IV		3	0.8	4	1.1	10	2.7	17	1.5	
unknown		170	45.5	130	34.8	75	20.1	375	33.4	
AJCC stage										
I		1	0.3	21	5.6	29	7.8	51	4.5	**< 0.001**
II		2	0.5	27	7.2	25	6.7	54	4.8	
III		0	0	10	2.7	12	3.2	22	2	
IV		1	0.5	12	3.2	7	1.9	20	1.8	
unknown		370	98.9	304	81.3	301	80.5	975	86.9	
Tumor size (cm)										
≤ 2		9	2.4	43	11.5	105	28.1	157	14	**< 0.001**
> 2 and ≤ 5		9	2.4	58	15.5	73	19.5	140	12.5	
> 5		1	0.3	29	7.8	27	7.2	57	5.1	
unknown		355	94.9	244	65.2	169	45.2	768	68.4	
LN status										
To be continued										
Negative		12	3.2	54	14.4	118	31.6	184	16.4	**< 0.001**
Positive		7	1.9	76	20.3	87	23.3	170	15.2	
unknown		355	94.9	244	65.2	169	45.2	768	68.4	
ER										
Negative		8	2.1	19	5.1	21	5.6	48	4.3	**< 0.001**
Positive		129	34.5	192	51.3	263	70.3	584	52.0	
unknown		237	63.4	163	43.6	90	24.1	490	43.7	
PR										
Negative		20	5.3	52	13.9	32	8.6	104	9.3	**< 0.001**
Positive		112	29.9	156	41.7	247	66.0	515	45.9	
unknown		242	64.7	166	44.4	95	25.4	503	44.8	
Her2										
Negative		3	0.8	55	14.7	61	16.3	119	10.6	**< 0.001**
Positive		1	0.3	10	2.7	8	2.1	19	1.7	
unknown		370	98.9	309	82.6	305	81.6	984	87.7	
Radiation										
Yes		92	24.6	88	23.5	75	20.1	255	22.7	**0.03**
No		282	75.4	284	75.9	292	78.1	858	76.5	
unknown		0	0	2	0.5	7	1.9	9	0.8	

*P*-value was calculated among all groups by the Chi-square test, and the bold type indicates significance. a: Including American Indian/Alaskan native, Asian/Pacific Islander and others-unspecified.

We also used the Cox proportional hazards model to investigate the effects of the clinical characteristics on OS in the matched group (Table 4). The univariate analysis results showed results similar to Table 2. In the multivariate analysis, white race could also be an independent protective factor when compared to the black race (HR = 1.153, 95% CI: 0.936–1.419, *P* = 0.001) and other races (HR = 1.447, 95% CI: 1.169–1.793, *P* = 0.003) (Table 4).

**Table 4:** Univariate and multivariate analysis of overall survival in the 1:1 matched groups

Variables	Univariate analysis		Multivariate analysis	
	HR (95% CI)	*P*-Value	HR (95% CI)	*P*-Value
Year of diagnosis				
1973–1993	reference		reference	
1994–2013	0.867 (0.740–1.015)	0.075	-	-
age (years)				
10–49	reference		reference	
50–85	2.619 (1.976–3.472)	**< 0.001**	2.741 (2.066–3.635)	**< 0.001**
Laterality				
right	reference		reference	
left	0.971 (0.836–1.128)	0.701	-	-
bilateral	1.220 (0.749–1.987)	0.425	-	-
Grade				
I	reference		reference	
II	1.183 (0.840–1.666)	0.336	1.300 (0.922–1.835)	0.135
III	1.740 (1.237–2.446)	**0.001**	1.687 (1.196–2.381)	**0.003**
IV	1.902 (1.037–3.491)	**0.038**	1.692 (0.911–3.142)	**0.096**
unknown	1.634 (1.179–2.266)	**0.003**	1.416 (1.008–1.988)	0.045
Tumor size (cm)				
≤ 2	reference		reference	
> 2 and ≤ 5	2.101 (1.305–3.383)	**0.002**	1.804 (1.118–2.912)	**0.016**
> 5	4.026 (2.346–6.909)	**< 0.001**	3.653 (2.112–6.318)	**0.003**
unknown	2.116 (1.441–3.109)	**< 0.001**	1.624 (1.084–2.434)	**0.019**
LN status				
Negative	reference		reference	
Positive	1.351 (0.904–2.018)	0.142	-	-
unknown	1.408 (1.029–1.926)	**0.033**	-	-
ER				
Negative	reference		reference	
Positive	0.865 (0.573–1.304)	0.488	4.006 (1.462–10.980)	**0.007**
unknown	1.204 (0.803–1.804)	0.369	3.244 (1.328–7.926)	**0.01**
PR				
Negative	reference		reference	
Positive	0.656 (0.498–0.866)	**0.003**	1.512 (0.630–3.627)	0.355
unknown	0.977 (0.752–1.269)	0.859	1.505 (0.668–3.390)	0.324
Race				
White	reference		reference	
Black	1.310 (1.105–1.552)	**0.002**	1.153 (0.936–1.419)	**0.001**
Other^b^	0.770 (0.632–0.937)	**0.009**	1.447 (1.169–1.793)	**0.003**

a: The total CI and *P*-value using Cox proportional hazards model and a bold type indicates significance. b: Including American Indian/Alaskan native, Asian/Pacific Islander.

### Stratification analysis with molecular subtype

To further investigate the effects of molecular subtypes on breast cancer outcomes between different races of patients, we stratified all the cases according to molecular subtype. In our study, only 1,796 cases had the definite subtype categorization when we eliminated all cases recorded before 2010. Hence, we attempted to conduct a subgroup analysis based on ER/PR/HER2 status. The results showed that 1,976 cases were included (1,748 cases of luminal, 15 cases of HER2+, and 33 cases of basal type). The subgroup distribution among whites, blacks, and others showed no significant difference (*P* = 0.475) (Table 5). We further performed the multivariate analysis, stratifying according to molecular subtype. However, all cases included were still alive during the follow-up period. Hence, we could not obtain more useful information for the subtype in the 3 races with MBC.

**Table 5:** Characteristics of patients with different ER/PR status

subtype	White(*n* = 1543)		Black (*n* = 293)		**other**^a^		total		
	No.	%	No.	%	No.	%	No.	%	*P*
Her+ 3	11	0.7	2	0.7	1	1.3	14	0.7	0.475
Luminal 1	1411	91.4	268	91.5	70	88.6	1749	91.3	
Basal 0	22	1.4	8	2.7	3	3.8	33	1.7	
Unknown 4	99	6.4	15	5.1	5	6.3	119	6.2	

*P*-value was calculated among all groups by the Chi-square test. a: Including American Indian/Alaskan native, Asian/Pacific Islander.

## DISCUSSION

Because of the delay in the diagnosis and loss of the social male-specific information, an increased trend in male breast cancer mortality rates has emerged. However, the relatively lower incidence of MBC than that of FBC has not aroused the same attention for improving research and prevention. At the present time, the management and treatment of MBC is based on guidelines developed for women [14]. It is known that FBC and MBC differ biologically. For example, the levels of hormone receptors in malignant tumors of the male mammary gland are higher than in malignant female breast tumors on average. The presence of receptor-positive tumors in men does not increase with the age, as is observed in FBC [15–17]. It is necessary to use optimized therapeutic approaches for the treatment of breast cancer in both sexes. Therefore, research on male breast cancer is needed to further promote treatment and prevention.

Because male breast cancer is a relatively rare disease, there is only limited data in the published literature regarding race as a risk factor in male breast cancer patients. For example, one report in 2011 showed the age-adjusted incidence rates overall and for white, black, and Hispanic males were 1.4, 1.3, 1.9, and 0.8 per 100,000, respectively [9, 18]. Crew *et al.* found that there was an association of black race with increased male breast cancer-specific mortality after adjustment for known clinical, demographic, and treatment factors using the SEER-Medicare database to identify men 65 years of age or older diagnosed with stage I-III breast cancer from 1991 to 2002 [19]. In our study, we obtained 8,607 cases from the current SEER database, and this study is currently the largest analysis of MBC in different races. The results provided evidence that white male breast cancer patients have a particular distribution of clinical characteristics. We summarized the clinicopathological characteristics of the 3 races with MBC and found that white patients presented with an older age, more were unmarried, they had smaller tumors, and they were more likely to be in stage I. Our study also indicated that the hormonal receptor-positive rate including ER, PR, and HER2 were higher in whites than in the blacks and others. Our study enrolled more cases of MBC from 1973 to 2013, and we analyzed more factors with racial disparities than Crew *et al.*

Common FBC risk factors such as the environment, genetics, hormones, smoking, and alcohol are also involved in the pathogenesis of male breast cancer [20]. For instance, one study found that MBC survival differences were observed between metropolitan and nonmetropolitan regions and an interaction between nonmetropolitan area and regional stage MBC was a significant predictor of poorer survival [21]. However, regional differences in tumor grade size and stage at diagnosis were not statistically significant. Only a small study analyzed those disparities in male breast cancer patients of different races. For example, Monederol *et al.* reported that smokers with male breast cancer had a significantly decreased survival rate [18].

This study provided some detailed relationships of these risk factors to race. The results showed that white and black male breast cancer patients have a poorer OS and BCSS than others. There were significant differences among whites, blacks, and others, such as the age of diagnosis (*P* < 0.001) and the hormone receptor status (ER, PR, and HER2, *P* < 0.001), which may be the main risk factors among whites, blacks and others.

Other factors might participate in the poorer OS of white and black patients than others. As a multifactorial disease, MBC requires a precise and comprehensive knowledge of the risk factors such as family history, genetic susceptibility, and predisposition for useful and effective treatment. In other words, male breast cancer can be affected by genetics, epigenetics, and ethical aspects [14]. In this study, we focused on the genetic and ethical factors to clarify the difference in clinical characteristics among the 3 groups. These might provide insights into a better understanding of MBC.

Our study has several limitations. FBC is categorized into different subtypes that have important prognostic implications, and clear racial/ethnic differences exist in the distribution of tumor subtypes [22–23]. However, it is not clear whether subtypes in MBC are associated with the same prognostic factors. It was reported that non-Hispanic blacks have more than triple the number of receptor-negative tumors and are more likely to have ER^+^/PR^−^ tumors than non-Hispanic black patients.[24] In our study, with the incomplete subtype data, we could not obtain more useful information for the subtype in the 3 races with MBC.

In conclusion, this study explored the clinicopathological characteristics and survival in white, black, and other races with male breast cancer, including American Indians, AK Natives, Asians, and Pacific Islanders. The white and black MBC patients have poorer OS and BCSS than the others. Race could also be a prognostic indicator. Differences in outcomes may be partially explained by the differences in tumor grade, LN status, and ER and PR status between the 3 groups. Our study might provide insights into a better understanding of MBC and further promote its treatment and prevention.

## MATERIALS AND METHODS

### Ethics statement

We obtained the SEER research data using the reference number 11443-Nov2015, and the data in the SEER database do not require informed patient consent. Our study was approved by the Ethical Committee and Institutional Review Board of Fudan University Shanghai Cancer Center (FDUSCC). The methods were performed in accordance with the approved guidelines.

### patients

The case listing in this study was generated by SEER *Stat version 8.3.2, which included data from 18 population-based registries (1973–2013) and covered approximately 28% of the United States. We choose 8,607 cases of patients according to the following criteria: male; known age; year of diagnosis before 2013; known race; unilateral breast cancer; pathologically confirmed breast cancer and breast cancer as the first and only malignant cancer diagnosis; known ER, PR, and HER2 status; and American Joint Committee on Cancer (AJCC) stages I-IV.

Patients were categorized according to the year of diagnosis (1973–2008 and 2009–2013), their ages (10–49 and 50–58 years), laterality (left or right or paired site), tumor size (tumor size ≤ 2 cm, tumor size 2–5 cm, or tumor size > 5 cm), LN, ER, PR, and HER2 status (negative, positive, and unknown), and radiation (yes, no, or unknown).

### Statistical analysis

The clinical characteristics of all selected cases were compared between different racial groups using the χ^2^ test. We used the Kaplan-Meier method to generate the survival curves, and the log-rank test was performed to compare the OS of white, black, and other (including American Indians, AK Natives, Asians, and Pacific Islanders) patients. OS was defined as the time from the date of diagnosis to the date of death due to all causes (including breast cancer) or the last follow-up. BCSS was measured from the date of diagnosis to the date of breast cancer death. Adjusted HRs with 95% CIs were calculated using Cox proportional hazard regression models to estimate the prognostic factors. These statistical analyses were performed utilizing SPSS software version 22.0. In addition, we matched white, black, and other male patients 1:1:1 on the following predetermined factors: age, AJCC stage, grade, breast subtype, utilizing psmatch 3.04 in SPSS designed for propensity score matching methods. In detail: binary treatment indicator: race; covariates: AJCC stage, grade, tumor size, LN status, AJCC stage, ER status, PR status, and HER2 status; matching algorithm: nearest neighbor matching; discard units outside of common support: none (always used by optimal matching); estimation algorithm: logistic regression; caliper: no caliper. A two-sided *P* value < 0.05 was considered statistically significant.

## SUPPLEMENTARY MATERIALS FIGURE



## Abbreviations

MBC: male breast cancer
FBC: female breast cancer
OS: overall survival
BCSS: breast cancer specific survival
CI: confidence interval
ER: oestrogen receptor
PR: progesterone receptor
HER2: human epidermal growth factor receptor 2
HR: hazard ratio
LN: lymph nodes
