# Supplementary material for: Clinicopathological characteristics and survival outcomes of male breast cancer according to race: A SEER population-based study
Source: Oncotarget. 2017 May 26;8(41):69680–90. doi: 10.18632/oncotarget.18265 (PMC5642508; doi:10.18632/oncotarget.18265)
Supplement: Supplementary file 1 [file oncotarget-08-69680-s001.pdf]

Clinicopathological characteristics and survival outcomes of male breast cancer according to race: A SEER population-based study

SUPPLEMENTARY MATERIALS

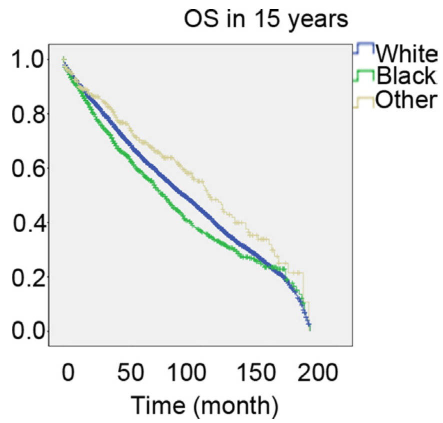

Supplementary Figure 1: The 15-year overall survival of White, Black and other patients. Kaplan meier test for 15-year overall survival (χ^2 =29.974, P < 0.001) to compare White patients to Blacks and others.
